# Assessment of the Association of Matrix Metalloproteinases with Myopia, Refractive Error and Ocular Biometric Measures in an Australian Cohort

**DOI:** 10.1371/journal.pone.0047181

**Published:** 2012-10-15

**Authors:** Maria Schache, Paul N. Baird

**Affiliations:** Centre for Eye Research Australia, University of Melbourne, Royal Victorian Eye and Ear Hospital, Melbourne, Australia; University of Regensburg, Germany

## Abstract

Extracellular matrix proteins have been implicated in protein remodelling of the sclera in refractive error. The matrix metalloproteinases (MMPs) falling into the collagenase (MMP1, MMP8, MMP13), gelatinase (MMP2, MMP9) and stromelysin (MMP3, MMP10, MMP11) functional groups are particularly important. We wished to assess their association with myopia, refractive error and ocular biometric measures in an Australian cohort. A total of 543 unrelated individuals of Caucasian ethnicity were genotyped including 269 myopes (≤−1.0D) and 274 controls (>−1.0D). Tag single nucleotide polymorphisms (SNPs) (n = 53) were chosen to encompass these eight MMPs. Association tests were performed using linear and logistic regression analysis with age and gender as covariates. Spherical equivalent, myopia, axial length, anterior chamber depth and corneal curvature were the phenotypes of interest. Initial findings indicated that the best p values for each trait were 0.02 for myopia at rs2274755 (*MMP9*), 0.02 for SE at both rs3740938 (*MMP8*) and rs131451 (*MMP11*), 0.01 for axial length at rs11225395 (*MMP8*), 0.01 for anterior chamber depth at rs498186 (*MMP1*) and 0.02 at rs10488 (*MMP1*). However, following correction for multiple testing, none of these SNPs remained statistically significant. Our data suggests that the MMPs in the collagenase, gelatinase and stromelysin categories do not appear to be associated with myopia, refractive error or ocular biometric measures in this cohort.

## Introduction

Refractive errors such as myopia are a group of common ocular disorders that result in blurred vision. The refractive status of the eye can be clinically defined using spherical equivalent (SE) measures and quantitated in dioptres (D). SE is commonly used, both in the clinic and academia, to define the overall refractive status of the eye and determine the nature and degree of refractive error [1]. The overall refractive status of the eye is influenced by a number of underlying components including ocular axial length, corneal curvature and lens thickness [2]. In particular, axial length is the most common factor associated with refractive error. Assessment of the determinants of refractive errors must not only include SE measures but also ocular biometric measures if we are to gain a better overall understanding of refractive error [3]. Unfortunately, often, such measures are lacking in many studies.

Biologically, the size and shape of the eye globe plays a key role in influencing the refractive status of the eye. In particular, an increase in ocular axial length can result in myopia and a reduction in scleral thickness [4]. This thinning of the sclera is not simply due to a passive stretching process but is the result of active remodelling of extracellular matrix components [5]. Scleral remodelling is a dynamic process resulting from an imbalance between the synthesis of extracellular matrix components such as collagens and proteoglycans and the degradation of extracellular matrix components by factors that include matrix metalloproteinases (MMPs).

MMPs are a group of zinc dependent endopeptidases that are involved in the degradation of extracellular matrix proteins. Depending on their substrate specificity, domain structure and cellular localisation, MMPs can be classified into collagenases (MMP1, MMP8, MMP13), gelatinases (MMP2, MMP9), stromelysins (MMP3, MMP10, MMP11), membrane-type (MMP14, MMP15, MMP16, MMP17, MMP24, MMP25) and matrilysins (MMP7, MMP26). Microarray based studies using healthy human donor scleral tissue have shown that multiple MMPs (MMP1–3, MMP7–17, MMP20, MMP24) are expressed suggesting that the scleral remodelling process in humans is, at least in part, driven by these MMPs [6]. Further evidence for the role of MMPs in scleral remodelling has come from animal studies.

Our understanding of the factors driving the scleral remodelling process has increased through the use of animal studies which have implicated the gelatinase, collagenase and stromelysin group of MMPs in myopia and refractive error. Animal studies typically induce myopia using form deprivation techniques that involve the application of monocular goggles that either restrict vision range or restrict clear vision. The scleral remodelling process in form deprivation myopia animal models results from an increase in collagen degradation and a decrease in collagen fibril diameter. In particular, collagen I, the major collagenous component of the mammalian sclera, has been shown to be selectively down regulated in tree shrew and chick models of form deprivation myopia [7]. This down regulation is the direct result of an increase in gelatinase A (MMP2) activity and may also be due to the activity of collagenases whose role is to cleave collagen I [8], [9]. In addition to collagen disturbances, scleral remodelling in form deprivation myopia results from an increase in proteoglycan turnover that is driven by the activities of gelatinase A and stromelysin [10]. For these reasons, MMPs are of interest in genetic association studies for myopia and refractive error.

The collagenase, gelatinase and stromelysin groups of MMPs have been partially analysed in genetic association studies for refractive error and myopia in human cohorts. In the case of refractive error, a recent study assessed, amongst others, these groups of MMPs, with the exception of *MMP11*, for association with refractive error in two family based cohorts, one of Ashkenazi Jewish origin and one Amish [11]. Positive associations were found in the Amish families with rs9928731 in *MMP2* (p = 00026) and rs1939008 (p = 00016) located in the intergenic region between *MMP1* and *MMP10*. These associations were not confirmed in the Ashkenazi Jewish families suggesting a potential founder effect. Thus, so far it is difficult to assess whether these findings are generalisable to non founder cohort groups in the wider population or only specific to certain groups. No other SNPs in this group of MMPs in these families showed positive associations with refractive error. In the case of high grade myopia there have been three studies, all in cohorts of Asian descent. These studies have used either a tag SNP approach in a single gene such as *MMP2* [12] or *MMP3* [13] or selected SNPs covering multiple MMPs such as *MMP1–3* [14] and have not reported positive associations. In the case of common myopia there has been one study in a Caucasian cohort that assessed selected SNPs in *MMP1*, *MMP3* and *MMP9* and found a positive association of myopia with the rs3025058 in *MMP3* (p = 0.015) and the rs17576(R279Q) in *MMP9* (p = 0.026) [15].

Despite many genetic studies analysing variants in the collagenase, stromelysin and gelatinase groups of MMPs, there is still more work that needs to be undertaken in order to have a comprehensive understanding of the role these genes play in refractive error and myopia. There are two areas, in particular, that need addressing. The first is an association study looking at ocular components that contribute to myopia such as axial length, corneal curvature and anterior chamber depth. These traits are yet to be examined in relation to the gelatinase, stromelysin and collagenase groups of MMPs. The second area is a replication study of the positive associations that have been detected so far. These are in *MMP2* for refractive error and in *MMP3* and *MMP9* for common myopia. Given this, our study ultimately aims to address these gaps in our current knowledge in order to both extend, as well as complement existing studies. The purpose of our study is to undertake a comprehensive genetic association study to assess the role of all the collagenase, gelatinase and stromelysin groups of MMPs in refractive error and myopia. An additional assessment as to the role that these MMPs might have in the endophenotypes of axial length, anterior chamber depth and corneal curvature will also be undertaken.

## Materials and Methods

### Subjects

Individuals were selected from the Genes in Myopia study with the recruitment process previously described [16], [17] [18]. Individuals were excluded based on four criteria (1) history of other eyes pathologies such as retinal detachment or keratoconus, (2) history of genetic disorders known to predispose to myopia, (3) anisometropia >2D difference between eyes and, (4) non-Caucasian ancestry.

Measurements for refraction (SE), axial length, corneal curvature and anterior chamber depth were taken for all individuals as described previously [16], [17]. For the current study myopia was defined as ≤−1.0D in the right eye. DNA from all consenting individuals was collected from venous blood samples [19]. Written informed consent was obtained from all individuals prior to any clinical examination, and ethics approval was provided by the Human Research and Ethics Committee of the Royal Victorian Eye and Ear Hospital, Melbourne. The study was conducted in accordance to the tenets of the Declaration of Helsinki.

### SNP selection and genotyping

Tag SNPs encompassing the coding region as well as 2 kb upstream of the start codon and 2 kb downstream of the stop codon of *MMP1*, *MMP2*, *MMP3*, *MMP8*, *MMP9*, *MMP10*, *MMP11*, *MMP13* were chosen. Methodology for choosing tag SNPs has been previously described [18]. Briefly, the Tagger section within the HaploView (version 4.2) software was used to identify tSNPs in these gene utilising a pairwise tagging approach, with the criteria of r^2^>0.8 and a minor allele frequency (MAF) >10% [20]. Tag SNPs were based on the CEU Hap Map population. All chosen SNPs were genotyped by the Australian Genome Research Facility (Melbourne, Australia) using Illumina® GoldenGate Genotyping assays and the Illumina iScan array scanner.

### Statistical analysis

Genotyping data were assessed for deviations from Hardy Weinberg equilibrium using PLINK (version 1.04) [21]. Any SNPs not passing this test in controls (P<0.05) were excluded from the analysis. Association tests were also performed using PLINK. Association tests for refraction (SE), axial length, corneal curvature and anterior chamber depth were performed using linear regression and tests for myopia were performed using logistic regression. All association tests included age and gender as covariates and adjustments for multiple testing using the Bonferroni correction were applied. Power calculations were performed using Quanto version 1.2.4 [22].

## Results

### Cohort Demographics

A total of 543 individuals were included in this study including 269 with myopia and 274 controls. For this study, myopia was defined as any individual with a SE ≤−1.0D and controls as those with SE >1.0D. This definition of myopia was used to reflect that used in the Hall *et al* study (2009) which is currently the only other study that assessed MMPs for an association with myopia. Refraction measures for the right and left eyes were highly correlated (r^2^>0.99) and thus only measures from the right eye were used in our analysis.

The mean age for the overall cohort was 50.9±14.9 years (49.9±16.1 years in the controls; 51.9±13.6 in the myopes). There were 35.8% males overall (31.1% in the controls; 40.7% in the myopes) and 64.2% females (69.0% in the controls; 59.3% in the myopes). A summary of all the clinical measures is shown in Table 1.

**Table 1:** Clinical characteristics of the study cohort for spherical equivalnet (SE), axial length (AL), corneal curvature (CC) and anterior chamber depth (ACD).

Phenotype	All	Controls	Cases
	Mean (SD)	Mean (SD)	Mean (SD)
SE	−1.8 (3.2)	0.7 (1.2)	−4.3 (2.6)
AL	24.3 (1.5)	23.5 (1.4)	25.1 (1.3)
CC	42.8 (2.8)	43.8 (1.8)	41.9 (3.3)
ACD	3.5 (0.4)	3.5 (0.3)	3.6 (0.4)

### Power calculation

Our power calculation showed that using a cohort size of 543 individuals (269 cases and 274 controls) has >80% power to detect an Odds Ratio of 1.8 assuming a minor allele frequency of 0.2 and an alpha of 0.001.

### Genetic association tests and power calculation

A total of 53 SNPs were genotyped including eleven in *MMP1*, ten in *MMP2*, three in *MMP3*, six in *MMP8*, three in *MMP9*, ten in *MMP10*, five in *MMP11* and five in *MMP13* (Table 2). The average SNP call rate was 97.62%. Three SNPs (rs11225426 in *MMP1*, rs7948454 in *MMP10* and rs3758854 in *MMP13*) were not in Hardy Weinberg Equilibrium and were therefore excluded from further analysis.The threshold for statistical significance for this study defined as P = 0.05/50 = 0.001.

**Table 2:** List of tagged SNPs genotyped for each matrix metalloproteinase gene.

Gene	Chromosome	Physical Location* (bp)	SNPs
*MMP1*	11	102,165,861–102,174,104	rs10488, rs11225426, rs1144393, rs2071232, rs3213460, rs470358, rs470504, rs470558, rs470747, rs498186, rs7125062
*MMP2*	16	54,070,589–54,098,103	rs1053605, rs11541998, rs11639960, rs11646643, rs1992116, rs243835, rs243840, rs243842, rs243866, rs7201
*MMP3*	11	102,211,738–102,219,552	rs3020919, rs522616, rs639752
*MMP8*	11	102,088,542–102,100,868	rs11225394, rs11225395, rs12284255, rs1320632, rs2012390, rs3740938
*MMP9*	20	44,070,954–44,078,606	rs17576, rs2274755, rs3918253
*MMP10*	11	102,146,444–102,156,554	rs12290253, rs17099562, rs17359286, rs3819099, rs4431992, rs470154, rs470171, rs486055, rs7119084, rs7948454
*MMP11*	22	22,445,036–22,456,502	rs131451, rs2267029, rs28382576, rs738791, rs738792
*MMP13*	11	102,318,935–102,331,672	rs11225490,rs17860584,rs3758854,rs478927

* HapMap Data Release 28 Phase2/3, August 2010.

Association tests for myopia using logistic regression indicated the best p-value (unadjusted) was 0.04 at rs28382576 for *MMP11* (Table 3). Association tests using linear regression analysis showed the best p-values (unadjusted) of 0.02 at rs3740938 in *MMP8* for spherical equivalent, 0.01 at rs11225395 in *MMP8* for axial length, 0.01 at rs498186 in *MMP1* for anterior chamber depth and 0.02 at rs10488 in *MMP1* for corneal curvature (Table 4). Following Bonferroni-correction, none of the SNPs retained statistical significance at a threshold of P<0.001.

**Table 3:** Tagged SNPs with the most associated p-values for each MMP gene using myopia as the trait.

SNP	Gene	Minor Allele	Cases		Controls		P*	OR	95% CI
			Freq.	HWE P	Freq.	HWE P			
rs470358	*MMP1*	A	0.42	0.90	0.40	0.10	0.51	1.09	0.85–1.40
rs243866	*MMP2*	A	0.23	0.86	0.26	0.43	0.28	0.85	0.64–1.14
rs639752	*MMP3*	A	0.52	0.71	0.48	0.33	0.18	1.18	0.93–1.50
rs3740938	*MMP8*	A	0.09	1.00	0.06	1.00	0.05	1.58	1.00–2.51
rs2274755	*MMP9*	A	0.20	0.33	0.14	0.81	0.02	1.48	1.06–2.06
rs17099562	*MMP10*	A	0.06	0.58	0.04	0.38	0.20	1.44	0.83–2.52
rs28382576	*MMP11*	A	0.07	1.00	0.04	1.00	0.04	1.84	1.03–3.26
rs478927	*MMP13*	A	0.36	0.50	0.30	0.77	0.07	1.27	0.98–1.64

* Unadjusted P-values with a significance threshold of 0.05/50 = 0.001.

**Table 4:** Tagged SNPs with the most associated p-values for each MMP gene using spherical equivalent (SE), axial length (AL), anterior chamber depth (ACD) and corneal curvature (CC) as the traits.

Gene	Trait	SNP	Minor Allele		HWE	P*	OR	95% CI
			Name	Freq.				
MMP1	SE	rs470747	G	0.36	0.93	0.10	1.40	0.93–2.10
	AL	rs10488	A	0.06	0.46	0.28	0.81	0.55–1.19
	ACD	rs498186	C	0.43	0.59	0.01	0.94	0.90–0.99
	CC	rs10488	A	0.06	0.46	0.02	2.40	1.17–4.92
MMP2	SE	rs11541998	C	0.11	0.13	0.17	1.52	0.83–2.77
	AL	rs11541998	C	0.11	0.13	0.25	0.83	0.61–1.13
	ACD	rs11541998	C	0.11	0.13	0.17	1.06	0.98–1.14
	CC	rs1053605	A	0.07	0.35	0.07	1.85	0.94–3.64
MMP3	SE	rs522616	G	0.21	0.19	0.37	1.23	0.78–1.96
	AL	rs522616	G	0.21	0.19	0.35	0.90	0.72–1.12
	ACD	rs3020919	A	0.24	0.29	0.15	0.96	0.91–1.02
	CC	rs3020919	A	0.24	0.29	0.51	1.15	0.75–1.76
MMP8	SE	rs3740938	A	0.08	1.00	0.02	0.42	0.20–0.85
	AL	rs11225395	A	0.46	0.93	0.01	1.28	1.05–1.54
	ACD	rs12284255	A	0.07	0.52	0.08	0.93	0.85–1.01
	CC	rs11225395	A	0.46	0.93	0.09	0.73	0.51–1.05
MMP9	SE	rs3918253	G	0.45	0.43	0.23	0.79	0.54–1.16
	AL	rs2274755	A	0.17	0.54	0.18	1.14	0.94–1.37
	ACD	rs3918253	G	0.45	0.43	0.43	0.97	0.91–1.04
	CC	rs3918253	G	0.45	0.43	0.30	0.83	0.58–1.18
MMP10	SE	rs470154	A	0.06	0.40	0.31	0.64	0.27–1.52
	AL	rs486055	A	0.15	0.87	0.06	1.29	0.99–1.69
	ACD	rs7948454	G	0.08	0.02	0.09	1.07	0.99–1.16
	CC	rs17099562	A	0.05	0.39	0.06	2.17	0.98–4.83
MMP11	SE	rs131451	G	0.12	0.29	0.02	0.50	0.28–0.90
	AL	rs738791	A	0.49	0.93	0.15	1.15	0.95–1.40
	ACD	rs738791	A	0.49	0.93	0.10	1.04	0.99–1.09
	CC	rs738792	G	0.11	0.51	0.31	0.75	0.43–1.30
MMP13	SE	rs478927	A	0.33	0.77	0.40	0.84	0.56–1.26
	AL	rs10502009	G	0.11	1.00	0.19	0.82	0.61–1.10
	ACD	rs3758854	A	0.07	0.01	0.29	1.05	0.96–1.15
	CC	rs10502009	G	0.11	1.00	0.21	1.42	0.82–2.48

* Unadjusted P-values with a significance threshold of 0.05/50 = 0.001.

## Discussion

Our study has undertaken a detailed genetic analysis into the association of the gelatinase, collagenase and stromelysin groups of MMPs in myopia and refractive error. More importantly this is the first study to assess associations of these MMPs in the ocular biometric measures of axial length, anterior chamber depth and corneal curvature. To date this is the most comprehensive study into this group of MMPs that has been undertaken whereas previous studies were more limited, in that only selected variants were chosen or there was no analysis of endophenotypes. Our methodological approach used a tag SNP strategy for association testing that allowed for complete genetic coverage of all known SNPs in the coding regions, intronic regions and 2 kb upstream of the start codon and 2 kb downstream of the stop codon of these MMPs. Using this approach, we were not able to detect any statistically significant association with the phenotypes analysed. There have been two SNPs rs3025058 (MMP3) and rs17576 (R279Q; MMP9) previously associated with myopia [15]. We directly genotyped rs17576 and were not able to confirm the association with common myopia in our cohort (Tables S1 and S2). In the case of rs3025058 we did not directly genotype this SNP. This SNP is physically located with the region tagged by our SNPs but there is no genotype information available from the Hap Map reference population (CEU) to enable assessment of the LD relationships between this SNP and those that we genotyped. Hence we cannot comment on weather the SNPs we genotyped will act as proxies for this SNP and it will have to be directly genotyped in order to confirm its lack of association with common myopia in our cohort.

Although our study did not indicate association of genetic variants with refractive error for these MMPs, there may be other extracellular matrix component genes that play a role. Extracellular matrix components such as collagen type I alpha 1 (*COL1A1*), collagen type II alpha 1 (*COL2A1*), lumican (*LUM*), decorin (*DCN*) and epiphycan (*EPYC* or *DSPG3*) have been previously assessed for genetic associations with myopia. These include assessment of *DSPG3* and *DCN* with high myopia but resulted in no association whereas assessment of *COL1A1*, *COL2A1* and *LUM* showed both positive and negative associations in different studies [23], [24], [25], [26], [27], [28], [29], [30], [31], [32], [33]. Of these genes only *COL2A1* has been assessed for associations with common myopia with a positive result being reported [34].

Evidence for genetic variants in extracellular matrix components as playing a role in refractive error is not strong so far. Most evidence has been derived from alterations in the expression of these genes where strong evidence from animal studies has shown that extracellular matrix components such as MMP2, proteoglycans and type I collagen are differentially expressed in form deprivation myopia where vision is modified using artificial lenses or translucent occludes. [7], [8], [35], [36]. These studies in combination with the lack of genetic association reported in our work suggest that single base changes in the primary DNA sequence, at least in these genes, are not the main driving force behind myopia in this cohort. This does not imply that these genes do not play a role in myopia in general but simply relates to the state of allele associations in the current study cohort. It should also be noted that the ability to detect an association in any population is dependent on the SNPs present which may in some cases account for lack of reproducibility between studies. In addition to gene expression changes it is also possible large structural variations, rather than single nucleotide polymorphisms in the DNA may play a role in myopia. These would include deletions, duplications and more complicated genomic rearrangements, and are commonly referred to as structural or copy number variations. In support of this, evidence has emerged that show copy number variations at chromosome Xq28 play a role in X-linked myopia that is associated with other clinical features such as color vision deficiencies [37]. Structural variations have been previously implicated in many other diseases such as age related macular degeneration [38], [39].

The study design used for this study has many strengths including the choice of cohort and the tag SNP approach. The cohort was chosen to include a homogenous population of Caucasian ancestry where we collected ancestry information from at least two generations. This allowed minimization of the potential effects of population admixture. The power calculation for this study suggests that the cohort size utilised is of sufficient size to detect modest genetic effects up to an odds ratio of 1.8. Although our cohort may potentially be too small to detect very small effects, the volume of the sample required to detect these effects exceeded the capacity of the recruitment processes of this study. However, our sample size is within the range of what has been previously reported which strengthens our justification for using a cohort of this size. In addition to this the reported relatively narrow confidence intervals also suggest that the study had suffient power to detect relavant association. The tag SNP approach is also important as it allows good coverage of all the SNPs in these genes. However, the tag SNP approach also has limitations in that in this study it only covered common SNPs with a minor allele frequency of 0.1 or greater and hence rare variants may be excluded. Additionally the tag SNP approach does not cover genetic variants such as epigenetic modification, copy number variations and structural variations that may influence the development of disease. Clearly, more work needs to be undertaken for these genes in order to gain a complete understanding of all the potential genetic variations that may contribute to the development of myopia.

Our study suggests that polymorphisms in MMP genes categorised as collagenases, stromelysins and gelatinases do not play a major role in refractive error, myopia, axial length, corneal curvature and anterior chamber depth. Although there is strong evidence that these genes are involved in the sclera remodelling process that accompanies myopia, we propose that their role is not driven by single nucleotide polymorphisms but instead is influenced by other genetic changes that might include copy number changes, epigenetic changes or alterations in the regulatory elements of the genes all of which may results in changes in expression.

## Supporting Information

Table S1 **Results for all tagged SNPs for each MMP using myopia as the trait.**
(DOCX)

Table S2 **Results for all tagged SNPs for each MMP using spherical equivalent (SE), axial length (AL), anterior chamber depth (ACD) and corneal curvature (CC) as the traits.**
(DOCX)

## References

[1] Biino G, Palmas MA, Corona C, Prodi D, Fanciulli M, et al (2005) Ocular refraction: heritability and genome-wide search for eye morphometry traits in an isolated Sardinian population. Hum Gen 116: 152–159. 10.1007/s00439-004-1231-6 15611866

[2] Young TL, Metlapally R, Shay AE (2007) Complex trait genetics of refractive error. Arch Ophthalmol 125: 38–48. 17210850 10.1001/archopht.125.1.38

[3] Baird PN, Schache M, Dirani M (2010) The GEnes in Myopia (GEM) study in understanding the aetiology of refractive errors. Progress in Retinal & Eye Research 31. 10.1016/j.preteyeres.2010.05.004 20576483

[4] Rada JA, Shelton S, Norton TT (2006) The sclera and myopia. Exp Eye Res 82: 185–200. 16202407 10.1016/j.exer.2005.08.009

[5] Guggenheim JA, McBrien NA (1996) Form-deprivation myopia induces activation of scleral matrix metalloproteinase-2 in tree shrew. Investigative Ophthalmology & Visual Science 37: 1380–1395. 8641841

[6] Young TL, Scavello GS, Paluru PC, Choi JD, Rappaport EF, et al (2004) Microarray analysis of gene expression in human donor sclera. Molecular Vision 10: 163–176. 15041956

[7] Gentle A, Liu Y, Martin JE, Conti GL, McBrien NA (2003) Collagen gene expression and the altered accumulation of scleral collagen during the development of high myopia. J Biol Chem 278: 16587–16594. 12606541 10.1074/jbc.M300970200

[8] Siegwart JT Jr, Norton TT (2005) Selective regulation of MMP and TIMP mRNA levels in tree shrew sclera during minus lens compensation and recovery. Investigative Ophthalmology & Visual Science 46: 3484–3492. 16186323 10.1167/iovs.05-0194 PMC1987367

[9] Schippert R, Brand C, Schaeffel F, Feldkaemper MP (2006) Changes in scleral MMP-2, TIMP-2 and TGFbeta-2 mRNA expression after imposed myopic and hyperopic defocus in chickens. Exp Eye Res 82: 710–719. 16289164 10.1016/j.exer.2005.09.010

[10] Rada JA, Nickla DL, Troilo D (2000) Decreased proteoglycan synthesis associated with form deprivation myopia in mature primate eyes. Investigative Ophthalmology & Visual Science 41: 2050–2058. 10892842

[11] Wojciechowski R, Bailey-Wilson JE, Stambolian D (2010) Association of matrix metalloproteinase gene polymorphisms with refractive error in Amish and Ashkenazi families. Investigative Ophthalmology & Visual Science 51: 4989–4995. 20484597 10.1167/iovs.10-5474 PMC3066601

[12] Leung KH, Yiu WC, Yap MK, Ng PW, Fung WY, et al (2011) Systematic investigation of the relationship between high myopia and polymorphisms of the MMP2, TIMP2, and TIMP3 genes by a DNA pooling approach. Investigative Ophthalmology & Visual Science 52: 3893–3900. 21421877 10.1167/iovs.11-7286

[13] Liang CL, Wang HS, Hung KS, Hsi E, Sun A, et al (2006) Evaluation of MMP3 and TIMP1 as candidate genes for high myopia in young Taiwanese men. Am J Ophthalmol 142: 518–520. 16935611 10.1016/j.ajo.2006.03.063

[14] Nakanishi H, Hayashi H, Yamada R, Yamashiro K, Nakata I, et al (2010) Single-nucleotide polymorphisms in the promoter region of matrix metalloproteinase-1, -2, and -3 in Japanese with high myopia. Investigative Ophthalmology & Visual Science 51: 4432–4436. 20435584 10.1167/iovs.09-4871

[15] Hall NF, Gale CR, Ye S, Martyn CN (2009) Myopia and polymorphisms in genes for matrix metalloproteinases. Investigative Ophthalmology & Visual Science 50: 2632–2636. 19279308 10.1167/iovs.08-2427

[16] Garoufalis P, Chen CYC, Dirani M, Couper TA, Taylor HR, et al (2005) Methodology and recruitment of probands and their families for the Genes in Myopia (GEM) Study. Ophthalmic Epidemiology 12: 383–392. 16283990 10.1080/09286580500281222

[17] Dirani M, Chamberlain M, Garoufalis P, Chen C, Guymer RH, et al (2008) Testing protocol and recruitment in the genes in myopia twin study. Ophthalmic Epidemiol 15: 140–147. 18569808 10.1080/09286580801939338

[18] Schache M, Chen CY, Dirani M, Baird PN (2009) The hepatocyte growth factor receptor (MET) gene is not associated with refractive error and ocular biometrics in a Caucasian population. Mol Vis 15: 2599–2605. 20011629 PMC2790478

[19] Richardson AJ, Narendran N, Guymer RH, Vu H, Baird PN (2006) Blood storage at 4 degrees C-factors involved in DNA yield and quality. J Lab Clin Med 147: 290–294. 16750666 10.1016/j.lab.2006.01.005

[20] Barrett JC, Fry B, Maller J, Daly MJ (2005) Haploview: analysis and visualization of LD and haplotype maps. Bioinformatics 21: 263–265. 15297300 10.1093/bioinformatics/bth457

[21] Purcell S, Neale B, Todd-Brown K, Thomas L, Ferreira MA, et al (2007) PLINK: a tool set for whole-genome association and population-based linkage analyses. Am J Hum Genet 81: 559–575 Epub 2007 Jul 2025. 17701901 10.1086/519795 PMC1950838

[22] Gauderman WJ, J.M M (2006) QUANTO 1.1: A computer program for power and sample size calculations for genetic-epidemiology studies. (http://hydrauscedu/gxe. Accessed 2012 September 20).

[23] Wang IJ, Chiang TH, Shih YF, Hsiao CK, Lu SC, et al (2006) The association of single nucleotide polymorphisms in the 5′-regulatory region of the lumican gene with susceptibility to high myopia in Taiwan. Mol Vis 12: 852–857. 16902402

[24] Chen ZT, Wang IJ, Shih YF, Lin LL (2009) The association of haplotype at the lumican gene with high myopia susceptibility in Taiwanese patients. Ophthalmology 116: 1920–1927. 19616852 10.1016/j.ophtha.2009.03.023

[25] Yip SP, Leung KH, Ng PW, Fung WY, Sham PC, et al (2011) Evaluation of proteoglycan gene polymorphisms as risk factors in the genetic susceptibility to high myopia. Investigative Ophthalmology & Visual Science 52: 6396–6403. 21743019 10.1167/iovs.11-7639

[26] Liang CL, Hung KS, Tsai YY, Chang W, Wang HS, et al (2007) Systematic assessment of the tagging polymorphisms of the COL1A1 gene for high myopia. J Hum Genet 52: 374–377. 17273809 10.1007/s10038-007-0117-6

[27] Inamori Y, Ota M, Inoko H, Okada E, Nishizaki R, et al (2007) The *COL1A1* gene and high myopia susceptibility in Japanese. Hum Gen June 8 Epub. 10.1007/s00439-007-0388-1 17557158

[28] Nakanishi H, Yamada R, Gotoh N, Hayashi H, Otani A, et al (2009) Absence of association between COL1A1 polymorphisms and high myopia in the Japanese population. Investigative Ophthalmology & Visual Science 50: 544–550. 18836165 10.1167/iovs.08-2425

[29] Metlapally R, Li YJ, Tran-Viet KN, Abbott D, Czaja GR, et al (2009) COL1A1 and COL2A1 genes and myopia susceptibility: evidence of association and suggestive linkage to the COL2A1 locus. Investigative Ophthalmology & Visual Science 50: 4080–4086. 19387081 10.1167/iovs.08-3346 PMC3936411

[30] Zhang D, Shi Y, Gong B, He F, Lu F, et al (2011) An association study of the COL1A1 gene and high myopia in a Han Chinese population. Mol Vis 17: 3379–3383. 22219633 PMC3247168

[31] Wang J, Wang P, Gao Y, Li S, Xiao X, et al (2012) High myopia is not associated with single nucleotide polymorphisms in the COL2A1 gene in the Chinese population. Mol Med Report 5: 133–137. 10.3892/mmr.2011.626 21993774

[32] Wang P, Li S, Xiao X, Jia X, Jiao X, et al (2009) High myopia is not associated with the SNPs in the TGIF, lumican, TGFB1, and HGF genes. Investigative Ophthalmology & Visual Science 50: 1546–1551. 19060265 10.1167/iovs.08-2537

[33] Majava M, Bishop PN, Hagg P, Scott PG, Rice A, et al (2006) Novel mutations in the small leucine-rich repeat protein/proteoglycan (SLRP) genes in high myopia. Hum Mutat 10.1002/humu.20444 17117407

[34] Mutti DO, Cooper ME, O'Brien S, Jones LA, Mariazita ML, et al (2007) Candidate gene and locus analysis of myopia. Mol Vis 13: 1012–1019. 17653045 PMC2776540

[35] Guggenheim JA, McBrien NA (1996) Form-deprivation myopia induces activation of scleral matrix metalloproteinase-2 in tree shrew. Investigative Ophthalmology & Visual Science 37: 1380–1395. 8641841

[36] Rada JA, Achen VR, Rada KG (1998) Proteoglycan turnover in the sclera of normal and experimentally myopic chick eyes. Investigative Ophthalmology & Visual Science 39: 1990–2002. 9761277

[37] Metlapally R, Michaelides M, Bulusu A, Li YJ, Schwartz M, et al (2009) Evaluation of the X-linked high-grade myopia locus (MYP1) with cone dysfunction and color vision deficiencies. Investigative Ophthalmology & Visual Science 50: 1552–1558. 19098318 10.1167/iovs.08-2455 PMC3934550

[38] Meyer KJ, Davis LK, Schindler EI, Beck JS, Rudd DS, et al (2011) Genome-wide analysis of copy number variants in age-related macular degeneration. Hum Genet 129: 91–100. 20981449 10.1007/s00439-010-0904-6 PMC3613489

[39] Neale BM, Fagerness J, Reynolds R, Sobrin L, Parker M, et al (2010) Genome-wide association study of advanced age-related macular degeneration identifies a role of the hepatic lipase gene (LIPC). Proc Natl Acad Sci U S A 107: 7395–7400. 20385826 10.1073/pnas.0912019107 PMC2867697

