# Supplementary material for: Assessment of the Association of Matrix Metalloproteinases with Myopia, Refractive Error and Ocular Biometric Measures in an Australian Cohort
Source: PLoS One. 2012 Oct 15;7(10):e47181. doi: 10.1371/journal.pone.0047181 (PMC3471969; doi:10.1371/journal.pone.0047181)
Supplement: Table S1 — Results for all tagged SNPs for each MMP using myopia as the trait. (DOCX) [file pone.0047181.s001.docx]

**Table S1: Results for all tagged SNPs for each MMP using myopia as the trait.**

| SNP | Gene | Minor | Cases | | Controls | | P | OR | 95% CI |
| --- | --- | --- | --- | --- | --- | --- | --- | --- | --- |
|  |  | Allele | Freq. | HWE P | Freq. | HWE P |  |  |  |
| rs10488 | MMP1 | A | 0.07 | 1.00 | 0.06 | 0.21 | 0.34 | 1.27 | 0.78 - 2.08 |
| rs11225426 | MMP1 | A | 0.09 | 0.00 | 0.12 | 0.02 | 0.25 | 0.81 | 0.56 - 1.17 |
| rs1144393 | MMP1 | G | 0.38 | 0.12 | 0.39 | 0.70 | 0.73 | 0.96 | 0.75 - 1.22 |
| rs2071232 | MMP1 | G | 0.21 | 0.05 | 0.20 | 1.00 | 0.70 | 1.06 | 0.79 - 1.43 |
| rs3213460 | MMP1 | A | 0.13 | 0.59 | 0.14 | 0.44 | 0.69 | 0.93 | 0.64 - 1.34 |
| rs470358 | MMP1 | A | 0.42 | 0.90 | 0.40 | 0.10 | 0.51 | 1.09 | 0.85 - 1.40 |
| rs470504 | MMP1 | A | 0.12 | 0.03 | 0.10 | 0.74 | 0.33 | 1.22 | 0.82 - 1.82 |
| rs470558 | MMP1 | A | 0.06 | 1.00 | 0.05 | 0.55 | 0.91 | 1.03 | 0.60 - 1.77 |
| rs470747 | MMP1 | G | 0.35 | 1.00 | 0.37 | 0.60 | 0.40 | 0.90 | 0.69 - 1.16 |
| rs498186 | MMP1 | C | 0.43 | 0.12 | 0.44 | 0.52 | 0.97 | 1.00 | 0.78 - 1.28 |
| rs7125062 | MMP1 | G | 0.30 | 0.11 | 0.30 | 1.00 | 0.84 | 1.03 | 0.79 - 1.33 |
| rs1053605 | MMP2 | A | 0.07 | 0.62 | 0.08 | 1.00 | 0.47 | 0.84 | 0.52 - 1.35 |
| rs11541998 | MMP2 | C | 0.11 | 0.05 | 0.12 | 0.77 | 0.60 | 0.90 | 0.62 - 1.32 |
| rs11639960 | MMP2 | G | 0.34 | 1.00 | 0.34 | 0.01 | 0.86 | 0.98 | 0.75 - 1.28 |
| rs11646643 | MMP2 | G | 0.35 | 0.28 | 0.35 | 0.11 | 0.81 | 0.97 | 0.74 - 1.26 |
| rs1992116 | MMP2 | A | 0.43 | 0.53 | 0.43 | 0.80 | 0.90 | 0.99 | 0.77 - 1.26 |
| rs243835 | MMP2 | A | 0.46 | 0.38 | 0.49 | 0.90 | 0.36 | 0.89 | 0.70 - 1.14 |
| rs243840 | MMP2 | G | 0.18 | 0.41 | 0.19 | 0.85 | 0.81 | 0.96 | 0.71 - 1.31 |
| rs243842 | MMP2 | G | 0.39 | 1.00 | 0.38 | 1.00 | 0.64 | 1.06 | 0.83 - 1.36 |
| rs243866 | MMP2 | A | 0.23 | 0.86 | 0.26 | 0.43 | 0.28 | 0.85 | 0.64 - 1.14 |
| rs7201 | MMP2 | C | 0.47 | 0.62 | 0.45 | 0.39 | 0.38 | 1.12 | 0.87 - 1.44 |
| rs3020919 | MMP3 | A | 0.26 | 0.63 | 0.23 | 0.39 | 0.28 | 1.17 | 0.88 - 1.57 |
| rs522616 | MMP3 | G | 0.21 | 0.45 | 0.22 | 0.29 | 0.63 | 0.93 | 0.70 - 1.24 |
| rs639752 | MMP3 | A | 0.52 | 0.71 | 0.48 | 0.33 | 0.18 | 1.18 | 0.93 - 1.50 |
| rs11225394 | MMP8 | A | 0.27 | 0.64 | 0.26 | 1.00 | 0.51 | 1.10 | 0.83 - 1.44 |
| rs11225395 | MMP8 | A | 0.47 | 1.00 | 0.44 | 0.90 | 0.27 | 1.15 | 0.90 - 1.47 |
| rs12284255 | MMP8 | A | 0.07 | 0.37 | 0.08 | 0.67 | 0.72 | 0.92 | 0.58 - 1.45 |
| rs1320632 | MMP8 | G | 0.09 | 1.00 | 0.09 | 1.00 | 0.86 | 0.96 | 0.63 - 1.48 |
| rs2012390 | MMP8 | G | 0.25 | 0.19 | 0.24 | 0.41 | 0.79 | 1.04 | 0.78 - 1.37 |
| rs3740938 | MMP8 | A | 0.09 | 1.00 | 0.06 | 1.00 | 0.05 | 1.58 | 1.00 - 2.51 |
| rs17576 | MMP9 | G | 0.40 | 0.61 | 0.36 | 0.69 | 0.09 | 1.25 | 0.97 - 1.60 |
| rs2274755 | MMP9 | A | 0.20 | 0.33 | 0.14 | 0.81 | 0.02 | 1.48 | 1.06 - 2.06 |
| rs3918253 | MMP9 | G | 0.47 | 0.46 | 0.43 | 0.62 | 0.23 | 1.16 | 0.91 - 1.47 |
| rs12290253 | MMP10 | G | 0.21 | 0.85 | 0.22 | 0.72 | 0.87 | 0.98 | 0.73 - 1.31 |
| rs17099562 | MMP10 | A | 0.06 | 0.58 | 0.04 | 0.38 | 0.20 | 1.44 | 0.83 - 2.52 |
| rs17359286 | MMP10 | A | 0.06 | 0.61 | 0.05 | 1.00 | 0.51 | 1.20 | 0.69 - 2.09 |
| rs3819099 | MMP10 | A | 0.14 | 1.00 | 0.15 | 0.47 | 0.56 | 0.90 | 0.63 - 1.28 |
| rs4431992 | MMP10 | G | 0.26 | 0.75 | 0.28 | 0.36 | 0.62 | 0.93 | 0.70 - 1.23 |
| rs470154 | MMP10 | A | 0.06 | 0.61 | 0.05 | 1.00 | 0.48 | 1.22 | 0.71 - 2.10 |
| rs470171 | MMP10 | C | 0.32 | 0.39 | 0.30 | 0.15 | 0.72 | 1.05 | 0.80 - 1.37 |
| rs486055 | MMP10 | A | 0.16 | 0.07 | 0.14 | 0.13 | 0.38 | 1.16 | 0.83 - 1.63 |
| rs7119084 | MMP10 | A | 0.22 | 0.72 | 0.21 | 0.20 | 0.55 | 1.10 | 0.81 - 1.48 |
| rs7948454 | MMP10 | G | 0.07 | 0.03 | 0.09 | 0.27 | 0.24 | 0.78 | 0.51 - 1.19 |
| rs131451 | MMP11 | G | 0.13 | 0.27 | 0.11 | 0.53 | 0.25 | 1.24 | 0.86 - 1.80 |
| rs2267029 | MMP11 | A | 0.08 | 1.00 | 0.08 | 0.41 | 0.78 | 1.07 | 0.69 - 1.65 |
| rs28382576 | MMP11 | A | 0.07 | 1.00 | 0.04 | 1.00 | 0.04 | 1.84 | 1.03 - 3.26 |
| rs738791 | MMP11 | A | 0.50 | 0.90 | 0.49 | 1.00 | 0.66 | 1.06 | 0.83 - 1.34 |
| rs738792 | MMP11 | G | 0.11 | 0.55 | 0.11 | 0.09 | 0.54 | 1.13 | 0.77 - 1.66 |
| rs10502009 | MMP13 | G | 0.11 | 1.00 | 0.11 | 1.00 | 0.97 | 1.01 | 0.69 - 1.48 |
| rs11225490 | MMP13 |  | NA | NA | NA | NA | NA | NA | NA |
| rs17860584 | MMP13 | G | 0.06 | 0.61 | 0.06 | 1.00 | 0.88 | 1.04 | 0.62 - 1.76 |
| rs3758854 | MMP13 | A | 0.08 | 0.06 | 0.07 | 0.10 | 0.36 | 1.23 | 0.79 - 1.92 |
| rs478927 | MMP13 | A | 0.36 | 0.50 | 0.30 | 0.77 | 0.07 | 1.27 | 0.98 - 1.64 |
