# Supplementary material for: Assessment of the Association of Matrix Metalloproteinases with Myopia, Refractive Error and Ocular Biometric Measures in an Australian Cohort
Source: PLoS One. 2012 Oct 15;7(10):e47181. doi: 10.1371/journal.pone.0047181 (PMC3471969; doi:10.1371/journal.pone.0047181)
Supplement: Table S2 — Results for all tagged SNPs for each MMP using spherical equivalent (SE), axial length (AL), anterior chamber depth (ACD) and corneal curvature (CC) as the traits. (DOCX) [file pone.0047181.s002.docx]

**Table S2: Results for all tagged SNPs for each MMP using spherical equivalent (SE), axial length (AL), anterior chamber depth (ACD) and corneal curvature (CC) as the traits.**

| SNP | Gene | Minor Allele | | HWE | Spherical Equivalent | | | Axial Length | | | Anterior Chamber Depth | | | Corneal Curvature | | |
| --- | --- | --- | --- | --- | --- | --- | --- | --- | --- | --- | --- | --- | --- | --- | --- | --- |
|  |  | Name | Freq. | P | P | OR | 95% CI | P | OR | 95% CI | P | OR | 95% CI | P | OR | 95% CI |
| rs10488 | MMP1 | A | 0.06 | 0.46 | 0.4235 | 0.73 | 0.33 - 1.59 | 0.2849 | 0.81 | 0.55 - 1.19 | 0.5649 | 1.03 | 0.93 - 1.13 | 0.01753 | 2.40 | 1.17 - 4.92 |
| rs11225426 | MMP1 | A | 0.10 | 0.00 | NA | NA | NA | NA | NA | NA | NA | NA | NA | NA | NA | NA |
| rs1144393 | MMP1 | G | 0.39 | 0.15 | 0.7674 | 1.06 | 0.72 - 1.56 | 0.6709 | 0.96 | 0.80 - 1.16 | 0.3489 | 1.02 | 0.98 - 1.07 | 0.624 | 1.09 | 0.76 - 1.57 |
| rs2071232 | MMP1 | G | 0.21 | 0.14 | 0.7249 | 0.92 | 0.57 - 1.48 | 0.8444 | 0.98 | 0.77 - 1.24 | 0.3416 | 0.97 | 0.92 - 1.03 | 0.9647 | 0.99 | 0.63 - 1.54 |
| rs3213460 | MMP1 | A | 0.13 | 0.26 | 0.6736 | 1.13 | 0.63 - 2.04 | 0.3758 | 0.88 | 0.66 - 1.17 | 0.7394 | 1.01 | 0.94 - 1.09 | 0.9181 | 0.97 | 0.56 - 1.67 |
| rs470358 | MMP1 | A | 0.41 | 0.33 | 0.2565 | 0.79 | 0.53 - 1.18 | 0.9945 | 1.00 | 0.82 - 1.22 | 0.4239 | 1.02 | 0.97 - 1.07 | 0.6832 | 0.93 | 0.64 - 1.34 |
| rs470504 | MMP1 | A | 0.11 | 0.18 | 0.4262 | 0.77 | 0.41 - 1.46 | 0.8266 | 1.04 | 0.76 - 1.42 | 0.277 | 1.05 | 0.97 - 1.13 | 0.5099 | 1.22 | 0.67 - 2.21 |
| rs470558 | MMP1 | A | 0.05 | 1.00 | 0.9543 | 0.98 | 0.41 - 2.30 | 0.4927 | 1.16 | 0.76 - 1.76 | 0.2296 | 1.07 | 0.96 - 1.18 | 0.9323 | 0.97 | 0.44 - 2.12 |
| rs470747 | MMP1 | G | 0.36 | 0.93 | 0.1033 | 1.40 | 0.93 - 2.10 | 0.8471 | 1.02 | 0.84 - 1.24 | 0.7168 | 0.99 | 0.94 - 1.04 | 0.923 | 1.02 | 0.70 - 1.49 |
| rs498186 | MMP1 | C | 0.43 | 0.59 | 0.9049 | 1.02 | 0.69 - 1.52 | 0.2975 | 0.90 | 0.74 - 1.10 | 0.0145 | 0.94 | 0.90 - 0.99 | 0.281 | 0.81 | 0.56 - 1.18 |
| rs7125062 | MMP1 | G | 0.30 | 0.22 | 0.5667 | 0.89 | 0.59 - 1.34 | 0.8587 | 0.98 | 0.80 - 1.20 | 0.8461 | 1.01 | 0.96 - 1.06 | 0.5229 | 1.13 | 0.77 - 1.66 |
| rs1053605 | MMP2 | A | 0.07 | 0.35 | 0.8914 | 1.05 | 0.50 - 2.24 | 0.9312 | 1.02 | 0.71 - 1.45 | 0.03282 | 1.10 | 1.01 - 1.21 | 0.07489 | 1.85 | 0.94 - 3.64 |
| rs11541998 | MMP2 | C | 0.11 | 0.13 | 0.1713 | 1.52 | 0.83 - 2.77 | 0.2492 | 0.83 | 0.61 - 1.13 | 0.168 | 1.06 | 0.98 - 1.14 | 0.1687 | 1.50 | 0.84 - 2.66 |
| rs11639960 | MMP2 | G | 0.34 | 0.07 | 0.5657 | 0.88 | 0.57 - 1.35 | 0.6827 | 1.04 | 0.85 - 1.29 | 0.5563 | 1.02 | 0.96 - 1.07 | 0.568 | 0.89 | 0.60 - 1.33 |
| rs11646643 | MMP2 | G | 0.35 | 0.06 | 0.8602 | 0.96 | 0.63 - 1.47 | 0.7721 | 1.03 | 0.84 - 1.27 | 0.8491 | 1.01 | 0.95 - 1.06 | 0.4219 | 0.85 | 0.58 - 1.26 |
| rs1992116 | MMP2 | A | 0.43 | 0.79 | 0.6016 | 0.90 | 0.61 - 1.33 | 0.7407 | 1.03 | 0.85 - 1.25 | 0.2311 | 1.03 | 0.98 - 1.08 | 0.99 | 1.00 | 0.70 - 1.43 |
| rs243835 | MMP2 | A | 0.47 | 0.49 | 0.3192 | 1.21 | 0.83 - 1.78 | 0.5987 | 0.95 | 0.79 - 1.15 | 0.2238 | 0.97 | 0.93 - 1.02 | 0.7999 | 0.96 | 0.67 - 1.36 |
| rs243840 | MMP2 | G | 0.19 | 0.78 | 0.6425 | 0.89 | 0.54 - 1.45 | 0.9371 | 0.99 | 0.78 - 1.26 | 0.588 | 1.02 | 0.96 - 1.08 | 0.2195 | 0.75 | 0.47 - 1.19 |
| rs243842 | MMP2 | G | 0.38 | 1.00 | 0.6073 | 1.11 | 0.75 - 1.65 | 0.8502 | 0.98 | 0.81 - 1.19 | 0.401 | 0.98 | 0.93 - 1.03 | 0.1988 | 1.28 | 0.88 - 1.86 |
| rs243866 | MMP2 | A | 0.24 | 0.81 | 0.2673 | 1.29 | 0.82 - 2.04 | 0.3404 | 0.90 | 0.72 - 1.12 | 0.1641 | 0.96 | 0.91 - 1.02 | 0.8646 | 1.04 | 0.68 - 1.57 |
| rs7201 | MMP2 | C | 0.46 | 0.30 | 0.2684 | 0.80 | 0.54 - 1.19 | 0.5145 | 1.07 | 0.88 - 1.30 | 0.4901 | 1.02 | 0.97 - 1.07 | 0.7517 | 1.06 | 0.73 - 1.54 |
| rs3020919 | MMP3 | A | 0.24 | 0.29 | 0.6671 | 0.90 | 0.57 - 1.43 | 0.2276 | 0.87 | 0.70 - 1.09 | 0.1527 | 0.96 | 0.91 - 1.02 | 0.5141 | 1.15 | 0.75 - 1.76 |
| rs522616 | MMP3 | G | 0.21 | 0.19 | 0.3722 | 1.23 | 0.78 - 1.96 | 0.3496 | 0.90 | 0.72 - 1.12 | 0.8929 | 1.00 | 0.94 - 1.05 | 0.8598 | 0.96 | 0.63 - 1.47 |
| rs639752 | MMP3 | A | 0.49 | 0.60 | 0.685 | 0.92 | 0.63 - 1.36 | 0.3724 | 0.92 | 0.76 - 1.11 | 0.6948 | 0.99 | 0.94 - 1.04 | 0.5649 | 0.90 | 0.63 - 1.29 |
| rs11225394 | MMP8 | A | 0.27 | 0.74 | 0.3166 | 0.80 | 0.52 - 1.24 | 0.02653 | 1.27 | 1.03 - 1.58 | 0.09611 | 1.05 | 0.99 - 1.10 | 0.5586 | 0.88 | 0.59 - 1.33 |
| rs11225395 | MMP8 | A | 0.46 | 0.93 | 0.07735 | 0.70 | 0.48 - 1.04 | 0.0126 | 1.28 | 1.05 - 1.54 | 0.1604 | 1.04 | 0.99 - 1.09 | 0.09484 | 0.73 | 0.51 - 1.05 |
| rs12284255 | MMP8 | A | 0.07 | 0.52 | 0.7627 | 1.12 | 0.54 - 2.31 | 0.4873 | 0.88 | 0.62 - 1.25 | 0.0834 | 0.93 | 0.85 - 1.01 | 0.321 | 1.40 | 0.72 - 2.71 |
| rs1320632 | MMP8 | G | 0.09 | 1.00 | 0.9021 | 0.96 | 0.48 - 1.90 | 0.8239 | 1.04 | 0.74 - 1.45 | 0.189 | 1.06 | 0.97 - 1.15 | 0.6092 | 0.85 | 0.45 - 1.60 |
| rs2012390 | MMP8 | G | 0.24 | 0.82 | 0.4507 | 0.84 | 0.54 - 1.32 | 0.6003 | 1.06 | 0.85 - 1.32 | 0.99 | 1.00 | 0.95 - 1.06 | 0.2773 | 0.80 | 0.53 - 1.20 |
| rs3740938 | MMP8 | A | 0.08 | 1.00 | 0.01698 | 0.42 | 0.20 - 0.85 | 0.2984 | 1.20 | 0.85 - 1.71 | 0.744 | 1.01 | 0.93 - 1.11 | 0.09814 | 0.57 | 0.29 - 1.11 |
| rs17576 | MMP9 | G | 0.38 | 1.00 | 0.3162 | 0.82 | 0.55 - 1.21 | 0.1487 | 1.15 | 0.95 - 1.40 | 0.7249 | 0.99 | 0.94 - 1.04 | 0.2408 | 0.80 | 0.56 - 1.16 |
| rs2274755 | MMP9 | A | 0.17 | 0.54 | 0.2406 | 0.73 | 0.43 - 1.23 | 0.1963 | 1.19 | 0.91 - 1.54 | 0.434 | 0.97 | 0.91 - 1.04 | 0.3251 | 0.78 | 0.47 - 1.28 |
| rs3918253 | MMP9 | G | 0.45 | 0.43 | 0.2339 | 0.79 | 0.54 - 1.16 | 0.1831 | 1.14 | 0.94 - 1.37 | 0.8628 | 1.00 | 0.96 - 1.05 | 0.3014 | 0.83 | 0.58 - 1.18 |
| rs12290253 | MMP10 | G | 0.21 | 0.70 | 0.6673 | 0.90 | 0.57 - 1.44 | 0.5551 | 0.93 | 0.74 - 1.18 | 0.3577 | 1.03 | 0.97 - 1.09 | 0.9259 | 1.02 | 0.66 - 1.58 |
| rs17099562 | MMP10 | A | 0.05 | 0.39 | 0.2258 | 0.58 | 0.24 - 1.40 | 0.9486 | 1.01 | 0.66 - 1.55 | 0.655 | 1.02 | 0.92 - 1.14 | 0.05709 | 2.17 | 0.98 - 4.83 |
| rs17359286 | MMP10 | A | 0.05 | 0.39 | 0.5009 | 0.74 | 0.31 - 1.78 | 0.5716 | 0.88 | 0.58 - 1.35 | 0.1961 | 1.07 | 0.96 - 1.20 | 0.9742 | 1.01 | 0.45 - 2.28 |
| rs3819099 | MMP10 | A | 0.14 | 0.60 | 0.6077 | 1.16 | 0.66 - 2.02 | 0.418 | 0.89 | 0.68 - 1.17 | 0.3161 | 1.04 | 0.97 - 1.11 | 0.7406 | 1.09 | 0.65 - 1.83 |
| rs4431992 | MMP10 | G | 0.27 | 0.38 | 0.8066 | 1.06 | 0.68 - 1.66 | 0.2426 | 0.88 | 0.71 - 1.09 | 0.3299 | 0.97 | 0.92 - 1.03 | 0.07881 | 1.45 | 0.96 - 2.18 |
| rs470154 | MMP10 | A | 0.06 | 0.40 | 0.3089 | 0.64 | 0.27 - 1.52 | 0.4132 | 1.19 | 0.78 - 1.82 | 0.5704 | 1.03 | 0.93 - 1.15 | 0.3833 | 0.70 | 0.32 - 1.55 |
| rs470171 | MMP10 | C | 0.31 | 0.76 | 0.4107 | 1.20 | 0.78 - 1.83 | 0.4331 | 1.09 | 0.88 - 1.34 | 0.6974 | 1.01 | 0.96 - 1.06 | 0.5082 | 0.87 | 0.59 - 1.30 |
| rs486055 | MMP10 | A | 0.15 | 0.87 | 0.4843 | 0.83 | 0.48 - 1.41 | 0.05997 | 1.29 | 0.99 - 1.69 | 0.1295 | 1.05 | 0.98 - 1.13 | 0.3647 | 0.79 | 0.47 - 1.32 |
| rs7119084 | MMP10 | A | 0.22 | 0.70 | 0.6335 | 0.89 | 0.55 - 1.44 | 0.4368 | 0.91 | 0.72 - 1.15 | 0.6294 | 0.99 | 0.93 - 1.05 | 0.2768 | 1.28 | 0.82 - 1.99 |
| rs7948454 | MMP10 | G | 0.08 | 0.02 | 0.5937 | 1.20 | 0.61 - 2.34 | 0.8991 | 0.98 | 0.71 - 1.35 | 0.08737 | 1.07 | 0.99 - 1.16 | 0.1875 | 1.50 | 0.82 - 2.76 |
| rs131451 | MMP11 | G | 0.12 | 0.29 | 0.02066 | 0.50 | 0.28 - 0.90 | 0.3048 | 1.16 | 0.87 - 1.54 | 0.5207 | 0.98 | 0.91 - 1.05 | 0.005257 | 0.47 | 0.28 - 0.80 |
| rs2267029 | MMP11 | A | 0.08 | 0.78 | 0.07 | 0.52 | 0.26 - 1.05 | 0.7418 | 1.06 | 0.76 - 1.48 | 0.6036 | 1.02 | 0.94 - 1.11 | 0.3275 | 0.73 | 0.39 - 1.37 |
| rs28382576 | MMP11 | A | 0.05 | 1.00 | 0.8715 | 0.93 | 0.39 - 2.24 | 0.6087 | 0.89 | 0.58 - 1.38 | 0.5458 | 0.97 | 0.87 - 1.08 | 0.3629 | 1.46 | 0.65 - 3.28 |
| rs738791 | MMP11 | A | 0.49 | 0.93 | 0.6392 | 1.10 | 0.74 - 1.62 | 0.1487 | 1.15 | 0.95 - 1.40 | 0.1018 | 1.04 | 0.99 - 1.09 | 0.941 | 1.01 | 0.70 - 1.46 |
| rs738792 | MMP11 | G | 0.11 | 0.51 | 0.04104 | 0.53 | 0.29 - 0.97 | 0.4106 | 1.13 | 0.84 - 1.52 | 0.3964 | 1.03 | 0.96 - 1.11 | 0.3081 | 0.75 | 0.43 - 1.30 |
| rs10502009 | MMP13 | G | 0.11 | 1.00 | 0.9109 | 1.04 | 0.56 - 1.91 | 0.1912 | 0.82 | 0.61 - 1.10 | 0.7019 | 1.01 | 0.94 - 1.09 | 0.2117 | 1.42 | 0.82 - 2.48 |
| rs11225490 | MMP13 | 0 | 0.00 | 1.00 | NA | NA | NA | NA | NA | NA | NA | NA | NA | NA | NA | NA |
| rs17860584 | MMP13 | G | 0.06 | 1.00 | 0.5361 | 1.30 | 0.56 - 3.00 | 0.5448 | 0.88 | 0.58 - 1.33 | 0.8953 | 1.01 | 0.91 - 1.12 | 0.8343 | 1.09 | 0.49 - 2.41 |
| rs3758854 | MMP13 | A | 0.07 | 0.01 | 0.4624 | 0.77 | 0.38 - 1.56 | 0.8766 | 1.03 | 0.72 - 1.46 | 0.2923 | 1.05 | 0.96 - 1.15 | 0.849 | 0.94 | 0.48 - 1.83 |
| rs478927 | MMP13 | A | 0.33 | 0.77 | 0.3976 | 0.84 | 0.56 - 1.26 | 0.4839 | 0.93 | 0.76 - 1.14 | 0.7182 | 1.01 | 0.96 - 1.06 | 0.8837 | 0.97 | 0.66 - 1.43 |
